# The Effects of Substituting Dietary Soybean Meal with Maize Grain on Milk Production in Dairy Goats

**DOI:** 10.3390/ani10020299

**Published:** 2020-02-13

**Authors:** Luca Rapetti, Gianluca Galassi, Andrea Rota Graziosi, Gianni Matteo Crovetto, Stefania Colombini

**Affiliations:** Dipartimento di Scienze Agrarie e Ambientali—Produzione, Territorio, Agroenergia, Università degli Studi di Milano, 20133 Milan, Italy; gianluca.galassi@unimi.it (G.G.); andrea.rota@unimi.it (A.R.G.); matteo.crovetto@unimi.it (G.M.C.); stefania.colombini@unimi.it (S.C.)

**Keywords:** dairy goat, protein content, goat milk, milk urea, soybean meal, maize grain

## Abstract

**Simple Summary:**

Today the environmental sustainability of livestock has become increasingly important. Nitrogen excretion from livestock can lead to air and groundwater pollution, causing acid rain, fine air particulates, and greenhouse gas emissions that lead to climate change, groundwater nitrate poisoning, and surface water eutrophication. Often, livestock are fed diets with a protein content that is much higher than what they require. In this experiment, a herd of dairy goats of the Alpine breed, reared on a farm in the hills near Genoa, Italy, was used to study the impacts of being fed a low- or a high-protein diet. The high-protein diet did not improve milk yield and quality; on the contrary, it lowered the efficiency of dietary-nitrogen utilization, increased feeding cost, and, above all, increased nitrogen excretion and the associated environmental risks. In conclusion, a high-protein diet is not advisable, both economically and environmentally, and a precision feeding approach (i.e., supplying the nutrients required for a given body mass and level of production, and no more) is highly recommended.

**Abstract:**

In view of better environmental sustainability, livestock diets must not exceed protein requirements, as often happens with lactating goats reared in semi-intensive systems. The aim of this experiment was to verify in real-breeding conditions the influence of two diets with different protein contents (% crude protein (CP) on dry matter (DM)): 16.0 (high-protein diet; HP) vs 12.2 (low-protein diet; LP) on milk production in dairy goats. The diets differed only in the replacement—in the LP diet—of 250 g soybean meal with 250 g maize grain meal. Twenty-three Alpine goats were divided into two groups and used in a cross-over feeding trial for 2 months. Animals were weighed at the beginning of each month of the trial, and feed intake and milk yield and composition were recorded weekly. HP and LP did not differ statistically for milk yield and composition (3.32 vs 3.42 kg milk/d, 3.21% vs 3.27% fat, 3.31% vs 3.27% protein for HP and LP, respectively), but the HP diet determined a higher milk urea content (51.2 vs 36.6 mg/dL, *p* < 0.001) and a worse efficiency of nitrogen utilization (28.0% vs 37.2%). In conclusion, the LP diet resulted in a reduction of urinary nitrogen excretion by 28% and of the feed cost by about 10%.

## 1. Introduction

Contemporary public opinion considers livestock, as one of the major components in the agricultural sector, to be largely responsible for the environmental damages our planet has, and continues to, experience. Despite this, several studies (among others [1]) have provided evidence for the lower impact of agriculture, and specifically, animal production, on the environment in comparison with other human activities. However, air and water pollution, together with the loss of forests and biodiversity, is certainly affected by animal husbandry. When considering climate change, probably the most worrying contemporary environmental issue, the livestock sector, with its 7.1 gigatons of CO_2_-eq emissions per year, is responsible for 14.5% of all human-induced greenhouse gas (GHG) emissions [2]. Therefore, animal production systems, which are important to the food supply worldwide, have to pay greater attention to being sustainable and environmentally friendly.

Among livestock farming, dairy goats have received an increased interest in economically developed countries because of the relatively low investment costs of this farming, its efficiency in terms of milk yield per unit of feed intake, and the growing appreciation of goats’ cheese by consumers. Goat farming systems in economically developed countries are normally intensive, with all the feed (forage and concentrate) being supplied in the manger, or are semi-intensive, with small pastures in addition to the feed supplied in the manger. Often, in these systems, most of the concentrate and part of the forage is purchased from the market, with a consequent imbalance between reared animals and available fields as well as a high stocking density, which can be harmful to the environment.

In particular, N excretion is normally high due to an excessive amount of protein supplied. This has been confirmed by a survey by Rapetti et al. [3], who reported that frequent high-milk urea contents in goat milk (>40 mg/dL) were associated with an excessive protein level in the diet. Urea is formed in the liver to detoxify the excess ammonia absorbed in the rumen in case of a surplus of nitrogenous substances (mainly protein), which is often associated with a lack of rapidly fermentable carbohydrates. Urea is then released from the liver in the bloodstream to reach, mainly, the kidneys and is excreted in the urine. However, a minor proportion of urea is excreted with milk or is recycled, via saliva, to reach the rumen. There is a highly positive correlation between blood urea and milk urea [4]; hence, the milk urea level (MUL) is a practical tool to estimate the protein balance of an animal, with high values indicating an excess of dietary protein, and very low values indicating a lack of it. Moreover, Giovanetti et al. [5] showed that the MUL is affected markedly not only by dietary protein, but also by dietary energy concentration.

According to Brun-Bellut et al. [6] an acceptable range for the MUL is 28–32, and according to Rapetti et al. [7], the acceptable range is 23–34 mg/dL. Indeed, considering the protein requirements of lactating goats, dairy goats can be satisfactorily fed diets with a moderate protein content. This has also been ascribed, at least in part, to the higher overall efficiency of goats, in comparison to cows, in transferring N from endogenous urea into milk [8], with consequently lower protein requirements in dairy goats as compared to dairy cows.

The frequently high crude protein (CP) content of dairy goat diets on intensive farms is due to the relatively high protein content of the forage in comparison with that normally fed to dairy cows, which is often based on maize silage, a forage that is very low in protein (7–8% on DM). For goats, milk production generally takes place in spring and summer, and the forages, both grazed or harvested as hay or silage, are normally above 12% CP on DM. Nevertheless, the feedstuff purchased is generally quite rich in protein (16–20% on DM), thus leading to diets that are redundant in nitrogen. Many farmers are still convinced that a generous protein supply can increase milk yield and quality. On the contrary, only the feed cost and environmental impact increase.

The aim of this study was to compare two diets with different protein contents in lactating goats, with the purpose of demonstrating that a diet with a moderate protein level, although sufficient to meet requirements, can be as effective as a diet with a high protein content in terms of milk production, and is more environmentally sustainable.

## 2. Materials and Methods

The experiment was conducted in April and May 2016 on a semi-intensive farm located in the Appenino, a province of Genoa, Italy. Twenty-three lactating Alpine goats were included, with a mean (±SD) body weight (BW) of 51.7 (±5.1) kg, 42 (±17) days in milk (DIM), and producing daily an average of 3.5 (±0.9) kg of milk at the start of the experiment. The goats were divided into two groups equal in BW, milk yield (MY), and DIM, and were then allocated randomly to one of two dietary treatments. The experiment was performed as a cross-over design, with inversion of the groups between the two treatments after one month (one month per period, with one week of diet adaptation and three weeks of data recording). The two experimental diets had an F:C ratio of about 55:45, and were characterized by different crude protein concentrations (HP, high-protein; LP, low-protein), but with a similar net-energy concentration obtained by substituting soybean meal with maize grain (Table 1). The goats were weighed at the beginning of each of the two experimental periods. The animals were fed daily with 1 kg/head of meadow hay (2nd cut), 750 g/head of a commercial concentrate mix (17% CP on DM), and with 500 g/head of a mix (1:1) of ground maize grain (9.4% CP on DM) and soybean meal (49.4% CP on DM—HP treatment) or 500 g/head of ground maize grain (LP treatment), for a total amount of 2 kg DM per goat. During the morning, for about four hours, all the goats had free access to a permanent pasture. The chemical composition of the pasture was not determined, but was assumed—on the basis of the tabulated data [9] of an upland permanent grassland—to be fresh forage (INRA code: FV0160) with 16.7% DM; 16.6% CP; 11.0% protein digestible in the intestine, with nitrogen as the limiting factor for rumen microbial growth (PDIN); 10.1% protein digestible in the intestine, with energy as the limiting factor for rumen microbial growth (PDIE); and 48.5% neutral detergent fibre (NDF) on DM. Although the assumption of a given analysis of pasture might introduce a bias in the evaluation of the composition of the diets effectively ingested, we believe that the difference between the two dietary treatments is not significantly influenced by this factor.

Table 1 reports an analysis of the ingredients supplied in the stall, while Table 2 shows the composition and the analysis of the two diets fed at the manger.

The predicted total voluntary-dry-matter intake (DMI, kg/d) was calculated according to Sauvant and Giger-Reverdin [11] as a function of BW (kg) and MY (kg/d), as follows: DMI = 0.257 + 0.0129 × BW + 0.405 × MY. The amount of dry matter ingested at grazing was estimated by the difference between the predicted DMI and the amount of DM ingested in the manger. The latter was considered equal to the amount supplied, since the feed refusals were always negligible.

During the two months of the trial, individual daily milk yield and group feed intake at the manger were registered weekly, with concomitant sampling for analyses.

The feed components of the diets fed at the manger were analyzed for the concentrations of ash (method 942.05) [12], CP (method 984.13) [12], ether extract (EE; method 920.29) [12], starch (method 996.11) [13], NDF corrected for insoluble ash and with the addition of α-amylase (aNDFom) [14], and ADF [15] using the Ankom 200 fiber apparatus (Ankom Technology Corp., Fairport, NY, USA). NFCs were calculated as a difference (NFC = 100 − (Ash + CP + EE + aNDFom)).

The following analyses were performed on the milk samples: urea was determined using the pH-differential technique [16]; somatic cells were counted (by Fossomatic 360, Foss, Hillerød, Denmark); and fat, crude protein, casein, and lactose levels were calculated (by Milkoscan FT6000, Foss, Hillerød, Denmark).

The requirements of intestinally digestible protein (PDI) were computed following Sauvant et al. [17]. In particular, the PDI requirement for maintenance was computed as a function of body weight (BW): PDI (g/d) = 0.62 BW (kg) + 12.8; the PDI requirement for milk production was calculated as a function of milk yield (MY) and milk true protein content (TP): PDI (g/d) = MY (kg/d) × TP (g/kg)/0.69, where TP was assumed to be 91.5% of milk crude protein. The concentrations of PDIN, PDIE, and NE_l_ were calculated using the equations reported by Baumont et al. [10]. Protein balance was computed as: (PDIN − PDI)/PDI × 100.

Statistical analysis was performed using the MIXED procedure of SAS [18]. Data were analyzed with the following model:

Y_ijk_ = μ + T_i_ + P_j_ + A_k_ + e_ijk_ (1)

where Y is the dependent variable calculated as the mean of three measurements (one per week) during each sampling period, μ is the overall mean, T_i_ is the treatment effect (i = 1, 2), P_j_ is the period effect (j = 1, 2), A_k_ is the animal random effect (k = 1, 23), and e_ijk_ is the residual error.

Least-squares mean estimates are reported. For all statistical analyses, significance was declared at *p* ≤ 0.05.

## 3. Results

The total dry-matter intake of the two groups was about 2.3 kg/d, consisting of 2.0 kg DM from the diet supplied at the manger and 0.3 kg DM predicted as pasture. Thus, the total CP, PDIN, and PDIE concentrations (% on DM) of the total diets ingested were 16.0%, 11.4%, and 11.1%, and 12.2%, 8.7%, and 9.6% for HP and LP, respectively.

The average performance results are reported in Table 3.

From these data (Table 3), it is evident that lowering the protein content of the diet by almost four percentage points (from 16.0% to 12.3% on DM) did not change the milk yield and composition, nor the somatic-cell count. On the contrary, the LP diet decreased the MUL significantly (from 51.2 to 36.6 mg/dL; *p* < 0.001). Regarding the milk fat content, results were equal to (for LP diet) or lower than (for HP diet) those for the protein content. This is rather common for goats reared in Northern Italy, as evidenced by Sandrucci et al. [19], who found that more than 50% of about 7000 goats monitored in their study had fat/protein reversions.

Considering the average estimated DM intake (2.3 kg/d), the LP and HP diets supplied a daily PDI of 200 and 255 g, respectively.

## 4. Discussion

The data obtained (Table 3) can be considered representative, on avearge, of the intensive goat farms in Northern Italy, both for milk yield and milk constituents. The results regarding milk production as a function of the dietary protein content are consistent with those of previous investigations. Shalu et al. [20] found that increasing the CP concentration of the diet from 13% to 17% did not improve MY nor milk protein content in dairy goats producing 3.65 kg/d. Similarly, Rapetti et al. [21] did not register any difference in MY (5.49 kg/d, on average) and in milk fat and protein in goats fed diets with 15.1% or 17.3% CP on DM in early lactation. In this study, the only difference was registered for the MUL, which was significantly lower in the low-protein diet. This is in agreement with the results obtained by Bonanno et al. [22] and Rapetti et al. [7], although the MUL values obtained in the present work are higher than the values predicted by the equations proposed by the two cited researchers. This gap is likely due to an underestimation of the real protein intake by the animals in the present work. However, the MUL difference between the two dietary treatments (14.6 mg/dL) is similar to those obtained by applying the prediction equations based on the CP concentration of the diet proposed by Bonanno et al. [22] (13.4 mg/dL) and Rapetti et al. [7] (18.4 mg/dL).

Considering the PDI balance, the value of 200 g/d supplied by the LP diet is in line with the requirements reported by Sauvant and Giger-Reverdin [11] for goats of this body weight and level of milk production. On the contrary, the PDI supplied (255 g/d) by the HP diet represented an excess of about 24% compared to the requirements.

Since the study was conducted on a commercial farm, the pasture intake could not be determined, but only estimated. However, it has to be underlined that this study was distinctive from other studies in that the proportion of diet derived from pasture was low (about 15% of total diet DM) and representative of a semi-intensive goat-rearing system typical of the area; hence, the results can be useful for farmers in order to improve the environmental and economic sustainability of the farm. As reported by Rapetti et al. [7], there is a lack of information and of consciousness regarding this matter, as demonstrated by the high MUL values (40 mg/dL, on average) of the bulk milk samples found in dairy goat farms in Northern Italy. Similarly, Sandrucci et al. [19] reported an average MUL (mg/dL) value of 39.7 in dairy goat farms in Northern Italy, with a wide variability among farms (from 11.4 to 90.9). The present results confirm that a diet that is too high in protein determines a very high urea content in the milk, which, in turn, is correlated with a high urinary N excretion that is detrimental to the environment due to the release of ammonia into the atmosphere.

The significantly lower MUL of the LP diet in comparison to the HP diet is probably due to the combined effects of a low N content and a high fermentable carbohydrate content (18.7% vs 26.9% starch on DM for the HP and LP diets, respectively) in comparison with fibrous carbohydrates. The latter enhances the process of microbial growth in the rumen, and, consequently, the use of ammonia to produce microbial protein, thus lowering the amount of ammonia absorbed through the rumen wall and reaching the liver through the bloodstream. Moreover, the efficiency of PDI utilization for the synthesis of milk protein (PDIeff = 0.66 × exp ^(−0.007 × (PDI − 100))^), according to the new INRA system for ruminants [23], should be 0.74 and 0.61 for LP and HP diets, respectively. This expected low efficiency of the HP diet could contribute to increasing the MUL, besides the effect due to the ammonia absorbed in the rumen.

Considering the efficiency of nitrogen utilization (milk N/intake N), the HP diet had an efficiency of 28%, which was lower than that computed for the LP diet (37.2%). This, in turn, was related to a higher urinary N (UN) excreted by the animals fed the HP diet in comparison to the LP diet. According to the equation for the prediction of UN excretion (g/d) in relation to the MUL (mg/dL) (UN = 0.513 × MUL − 0.125) proposed by Rapetti et al. [7], decreasing the dietary protein content in this experiment allowed for a 28% reduction in urinary N excretion. Similarly, applying the equation proposed by Sauvant et al. [24], the UN (g/d/kg BW) estimated from milk urea nitrogen (MUN, g/L) (UN = 0.078 + 1.06 × MUN) decreased by 22% with the LP diet.

Finally, the reduction of the dietary-protein content allowed a reduction of about 10% in feed costs.

## 5. Conclusions

The results obtained in the trial suggest the economic and environmental benefits and convenience of using diets with a moderate protein content. A protein content higher than the requirements does not give any advantage in terms of milk production, and it determines a high N excretion along with the related environmental problems.

## Figures and Tables

**Table 1:** Chemical composition and nutritive value of the feed components of the two experimental diets fed at the manger.

Item		Meadow Hay	Ground Maize Grain	Soybean Meal	Mixed Concentrate ^1^
Ash	% on DM	8.9	1.4	7.4	9.2
CP	% on DM	8.0	9.4	49.4	17.3
PDIN ^2^	% on DM	5.2	7.4	36.0	13.1
PDIE ^3^	% on DM	7.0	9.7	25.3	12.7
EE	% on DM	1.7	4.3	1.9	6.8
aNDFom	% on DM	60.8	12.0	14.2	23.5
ADF	% on DM	37.6	3.0	8.5	13.7
NFC ^4^	% on DM	20.6	72.9	27.1	42.4
NEl ^5^	Mcal/kg DM	1.07	2.07	2.04	1.88

^1^ Mixed concentrate made of (% on DM): 37.4 ground maize grain, 21.6 whole toasted soybean seeds, 17.1 soybean hulls, 7.0 barley meal, 5.4 partially dehulled solvent sunflower meal, 4.0 dehydrated lucerne meal, 2.3 sugarcane molasses, and 5.2 mineral/vitamin supplement. ^2^ PDIN: protein digestible in the intestine, with nitrogen as the limiting factor for rumen microbial growth, calculated according to Baumont et al. [10]. ^3^ PDIE: protein digestible in the intestine, with energy as the limiting factor for rumen microbial growth, calculated according to Baumont et al. [10]. ^4^ NFC: non-fibrous carbohydrates, calculated as follows: NFC = 100 − (Ash + CP + EE + aNDFom). ^5^ NEl: net energy for lactation.

**Table 2:** Composition and chemical analysis of the two experimental diets fed at the manger with high-protein (HP) or low-protein (LP) contents.

		HP	LP
Composition			
Meadow hay	kg/d	1.00	1.00
Mixed concentrate	kg/d	0.75	0.75
Ground maize grain	kg/d	0.25	0.50
Soybean meal (46% CP)	kg/d	0.25	-
Analysis			
Ash	% on DM	8.0	7.4
CP	% on DM	15.8	11.4
PDIN ^1^	% on DM	11.4	8.3
PDIE ^2^	% on DM	11.2	9.5
EE	% on DM	3.7	4.0
aNDFom	% on DM	37.9	37.7
ADF	% on DM	22.7	22.1
Starch	% on DM	18.7	26.9
NFC ^3^	% on DM	34.3	39.3
NEl ^4^	Mcal/kg DM	1.56	1.56

^1^ PDIN: protein digestible in the intestine, with nitrogen as the limiting factor for rumen microbial growth. ^2^ PDIE: protein digestible in the intestine, with energy as the limiting factor for rumen microbial growth. ^3^ NFC: non-fibrous carbohydrates, calculated as follows: NFC = 100 – (Ash + CP + EE + aNDFom). ^4^ NEl: net energy for lactation.

**Table 3:** Milk yield and composition of the goats fed high-protein (HP) or low-protein (LP) diets.

Item		HP	LP	SE	*p*-Value
Body weight	kg	51.4	51.1	1.30	0.896
Milk	kg/d	3.32	3.43	0.16	0.385
FPCM ^1^	kg/d	3.08	3.24	0.15	0.172
Fat	g/100 g	3.21	3.27	0.08	0.238
Crude Protein	g/100 g	3.31	3.27	0.05	0.136
Casein	g/100 g	2.41	2.38	0.04	0.126
Lactose	g/100 g	4.59	4.60	0.04	0.754
Urea	mg/dL	51.2	36.6	0.87	<0.001
LS ^2^		4.41	4.21	0.27	0.281

^1^ FPCM: milk corrected for fat (3.5%) and true protein (3.1%) derived from the indications of the French system [17], as follows: FPCM = Milk × (0.1375 × Fat% + 0.0825 × True Protein% + 0.263). ^2^ LS: linear score (LS = log_2_ somatic-cell count/12,500).
